# Gitelman Syndrome Presenting With Seizures and Atypical Features: A Case Series

**DOI:** 10.7759/cureus.87840

**Published:** 2025-07-13

**Authors:** Baiju Faizal Puthenkote, Monica Jadhav, Sahithi Surapaneni, Yogesh Yadav, Abdul Manaf

**Affiliations:** 1 Internal Medicine, Lifecare Hospital Musaffah, Abu Dhabi, ARE; 2 Research and Health Innovation, Lifecare Hospital Musaffah, Abu Dhabi, ARE; 3 Laboratory, Lifecare Hospital Musaffah, Abu Dhabi, ARE; 4 Radiology, Lifecare Hospital Musaffah, Abu Dhabi, ARE

**Keywords:** basal ganglia calcification, case series, gitelman syndrome, hypocalcemia, hypomagnesemia, hyponatremia, secondary hyperparathyroidism, seizures

## Abstract

Gitelman syndrome (GS) is a rare autosomal recessive salt-losing tubulopathy caused by mutations in the *SLC12A3* gene, leading to hypokalemia, hypomagnesemia, hypocalciuria, and metabolic alkalosis. We present a case series of two patients with GS, both presenting with seizures, a rare manifestation, alongside distinct atypical features. Case 1, a 35-year-old male, exhibited tetanic spasm, hypocalcemia, secondary hyperparathyroidism, and basal ganglia calcification. Case 2, a 55-year-old male with a history of coronary artery disease, presented with seizure, hypomagnesemia, and hyponatremia. These cases highlight the clinical heterogeneity of GS, emphasizing the importance of considering atypical presentations and their implications for diagnosis and management.

## Introduction

Gitelman syndrome (GS) is a rare autosomal recessive renal tubular disorder first described in 1966 by Gitelman et al., characterized by hypokalemia, hypomagnesemia, metabolic alkalosis, and hypocalciuria due to dysfunction of the thiazide-sensitive sodium-chloride cotransporter (NCC) in the distal convoluted tubule [1]. Mutations in the SLC12A3 gene, which encodes this NCC, lead to impaired sodium and chloride reabsorption, resulting in chronic renal salt wasting and compensatory secondary hyperaldosteronism [2].

The clinical presentation of GS is heterogeneous and often subtle, typically manifesting during adolescence or early adulthood. Common symptoms include fatigue, muscle cramps, salt cravings, and paresthesias. More severe cases may feature tetany, carpopedal spasms, and growth retardation in children. While biochemical abnormalities are often striking, the symptom burden may be underestimated, and health-related quality of life can be significantly impaired [3]. Seizures are a rarely reported manifestation of GS [4], often secondary to profound hypomagnesemia or associated disturbances in calcium homeostasis. Infrequent and atypical features such as hypocalcemia, secondary hyperparathyroidism, or basal ganglia calcification further complicate the clinical picture and may obscure diagnosis. Additionally, neurological presentations including encephalopathy, dizziness, and, in extreme cases, calcifications in deep grey matter nuclei have occasionally been linked to electrolyte imbalances associated with GS.

Here, we present a case series of two adult male patients diagnosed with GS, both of whom presented with seizures. One case was further complicated by systemic mineral dysregulation, including secondary hyperparathyroidism and basal ganglia calcification, thereby highlighting the diverse and atypical phenotypic spectrum of GS. These cases emphasize the importance of maintaining a high index of suspicion in patients with unexplained electrolyte abnormalities and neuro-muscular symptoms [4].

## Case presentation

Case 1

A 35-year-old male, Indian national, manual laborer by profession, presented to the emergency department with a two-year history of recurrent tetanic seizures characterized by carpopedal spasms and muscle stiffness. He reported chronic fatigue and occasional muscle cramps but denied polyuria or polydipsia. His medical history was unremarkable, with no family history of renal or neurological disorders. He worked on a construction site with limited sun exposure due to protective clothing and was not taking diuretics. He was taking phenytoin with a prior “seizure” disorder diagnosis.

Examination

The patient was conscious, alert, and oriented, with no neurological deficits. Blood pressure was 110/70 mmHg without postural hypotension, pulse was 88 bpm (regular), and Chvostek’s sign and Trousseau's sign were positive.

Laboratory Findings

Routine laboratory tests are given in Table 1.

**Table 1:** Laboratory investigations for case 1

Test	Result	Reference range	Interpretation
Serum potassium (K⁺)	2.7 mmol/L	3.7–5.5 mmol/L	Low (hypokalemia)
Serum magnesium (Mg²⁺)	0.63 mmol/L	0.66–1.07 mmol/L	Low (hypomagnesemia)
Corrected serum calcium (Ca²⁺)	1.20 mmol/L	2.15–2.5 mmol/L	Very Low (severe hypocalcemia)
Serum phosphorus (PO₄³⁻)	1.86 mmol/L	0.81–1.45 mmol/L	High (hyperphosphatemia)
Serum bicarbonate (HCO₃⁻)	31 mmol/L	22–28 mmol/L	High (metabolic alkalosis/compensation)
Arterial pH	7.40	7.35–7.45	Normal
24-hour urine calcium	2.34 mmol/day	2.5–8.0 mmol/day	Low
Serum uric acid	137 µmol/L	202–416 µmol/L	Low
25-hydroxyvitamin D	15 ng/mL	30–80 ng/mL	Low (vitamin D deficiency)
Parathyroid hormone	21.6 pmol/L	1.6–6.9 pmol/L	High (secondary hyperparathyroidism)
Serum creatinine	61 µmol/L	62–106 µmol/L	Low-normal
Creatine phosphokinase (CPK/CK)	6,242 U/L	0–190 U/L	Very high
ECG QTc interval	477 ms	<450 ms (males), <470 ms (females)	Prolonged QT interval

Imaging

**Figure 1:**
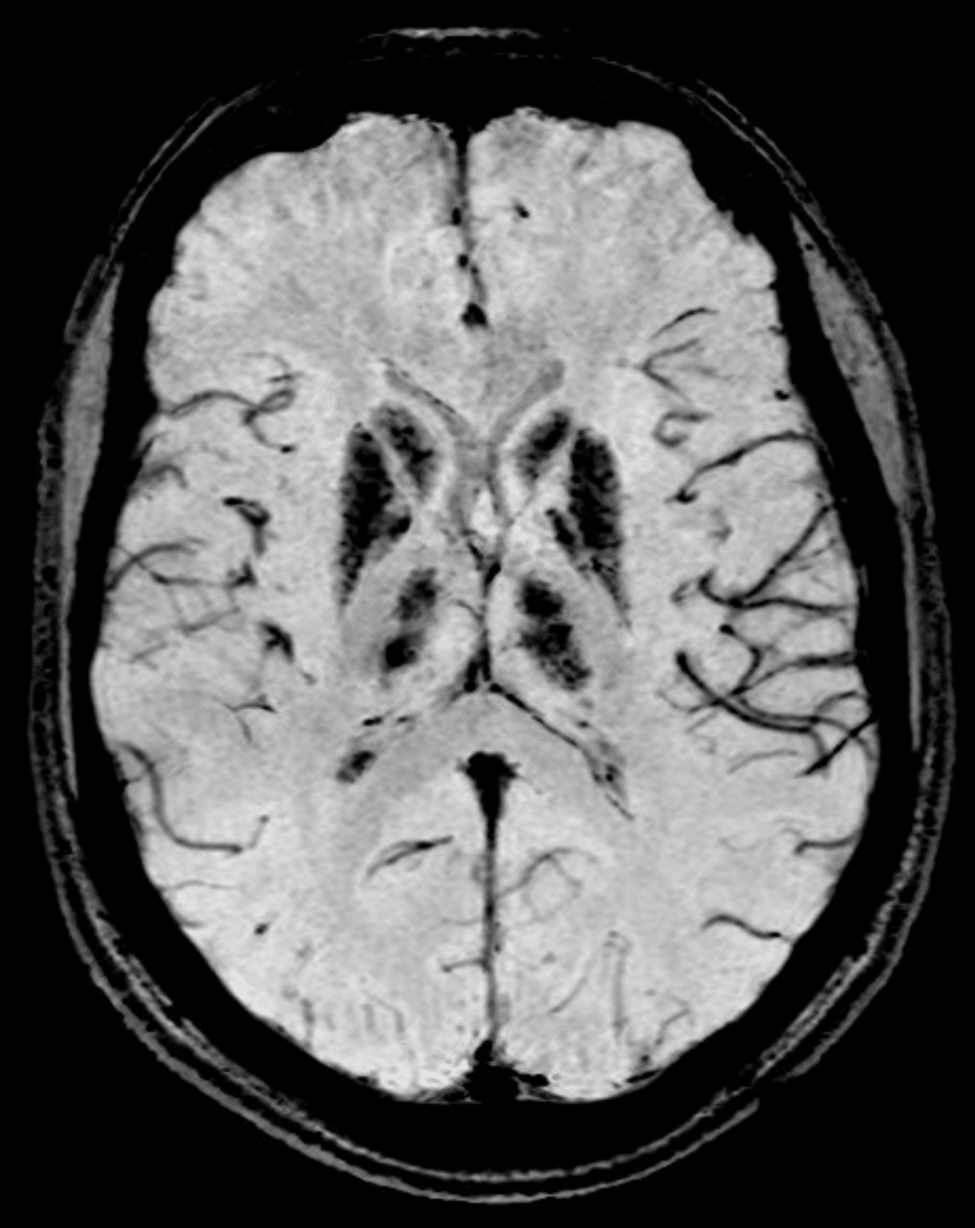
SWI (susceptibility-weighted imaging) axial MRI of the brain (plain study) image at the level of basal ganglia showing blooming in the bilateral basal ganglia, with both thalami signifying calcification

**Figure 2:**
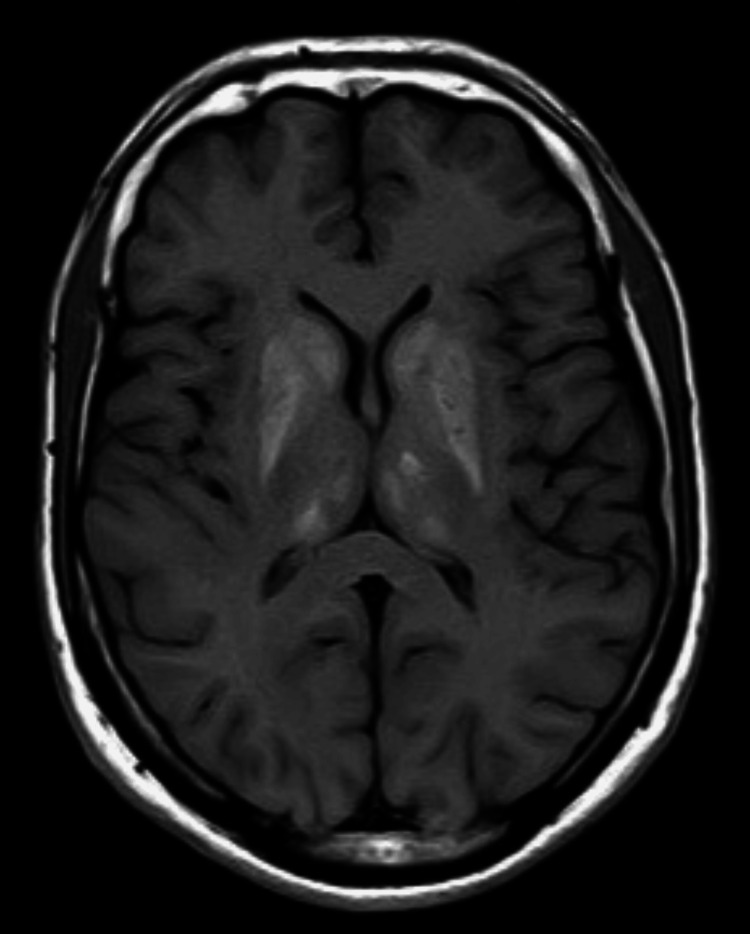
T1-weighted axial MRI of the brain (plain study) image at the level of basal ganglia showing hyperintense signal in the bilateral basal ganglia, with both thalami suggestive of calcification

Brain MRI revealed bilateral, symmetrical high T1 signal and iso-to-low T2 signal with dropout on gradient echo in the caudate, lentiform nuclei, thalami, and dentate nuclei, suggestive of calcification (Figures 1, 2). Renal ultrasound was normal.

Other Tests

**Figure 3:**
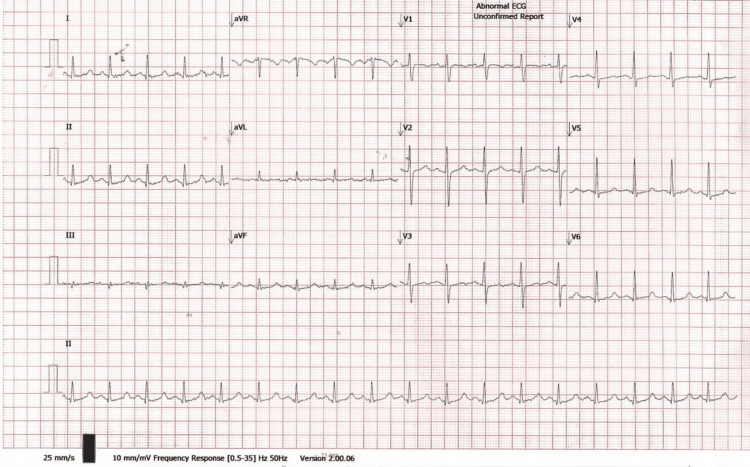
ECG all leads showing QTc 477 ms

Plasma renin activity was 8.2 ng/mL/h (0.5-3.5 ng/mL/h), and aldosterone was 28 ng/dL (4-21 ng/dL). ECG showed sinus rhythm with prolonged QTc interval (Figure 3). Genetic testing failed to identify homozygous *SLC12A3* mutations, though GS was clinically diagnosed. Workup for malabsorption (stool fat, stool elastase, serum tissue transglutaminase-immunoglobulin G [tTG-IgG], tissue transglutaminase-immunoglobulin A [tTG-IgA]) was negative.

Treatment

Magnesium and potassium supplementation, along with vitamin D and calcium, in case 1 resolved symptoms rapidly. Antiepileptics were tapered off and stopped.

Case 2

A 55-year-old male, Indian national, with a history of coronary artery disease (post-angioplasty in 2018) presented with a 1-minute loss of consciousness witnessed by a friend. He reported polyuria and nocturia for one year but denied persistent vomiting, diarrhea, or diuretic use. Medications (aspirin, atorvastatin, olmesartan) were stopped by him three months post-angioplasty.

Examination

The patient was conscious and oriented with no neurological deficits. A tongue bite injury was noted. He was vitally stable except for tachycardia.

Laboratory Findings

Routine laboratory test findings are given in Table 2.

**Table 2:** Laboratory investigations for case 2

Test	Result	Reference Range	Interpretation
Serum sodium (Na⁺)	129 mEq/L	135–145 mEq/L	Low (hyponatremia)
Serum potassium (K⁺)	3.1 mEq/L	3.7–5.5 mEq/L	Low (hypokalemia)
Serum ionized calcium (Ca²⁺)	1.02 mmol/L	1.15–1.35 mmol/L	Low (ionized hypocalcemia)
Serum total calcium	2.30 mmol/L	2.15–2.5 mmol/L	Normal (but ionized calcium is low)
Serum magnesium (Mg²⁺)	0.46 mmol/L	0.66–1.07 mmol/L	Very low (significant hypomagnesemia)
Serum osmolality	271 mOsm/kg	275–295 mOsm/kg	Low (hypo-osmolality)
Venous blood gas pH	7.55	7.31–7.41 (venous)	High (alkalemia)
Venous bicarbonate (HCO₃⁻)	33 mEq/L	22–28 mEq/L	High (metabolic alkalosis)
Venous PCO₂	42 mmHg	40–50 mmHg (venous)	Normal
Venous PO₂	70 mmHg	30–50 mmHg (venous)	Slightly high for venous
24-hour urine calcium	31 mg/L	100–300 mg/L	Low (hypocalciuria)
Serum creatinine	72 µmol/L	62–106 µmol/L	Normal
Thyroid-stimulating hormone	1.5 mIU/L	0.4–4.5 mIU/L	Normal

Imaging

**Figure 4:**
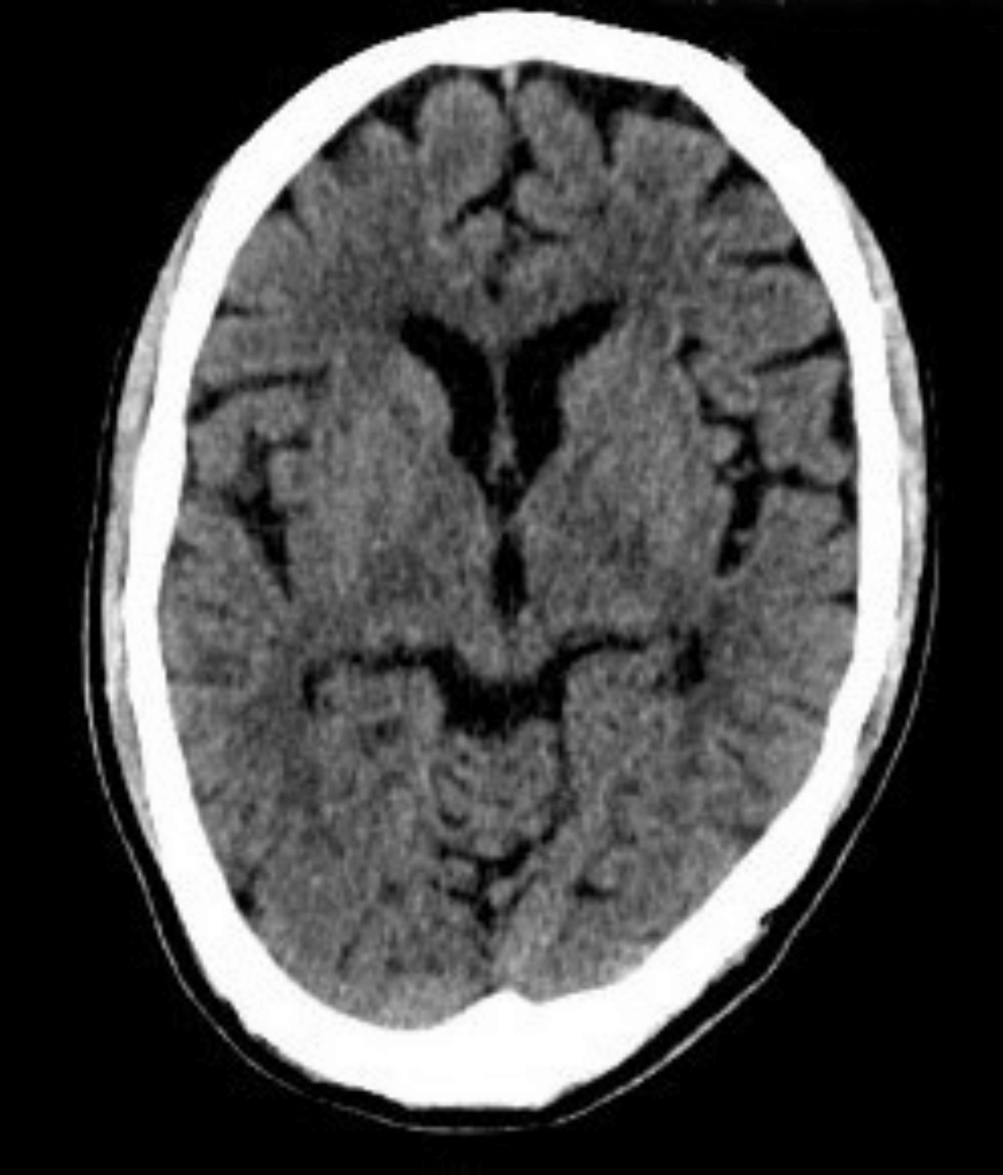
CT scan of the brain (axial section) at the level of basal ganglia (plain study) not showing any abnormality

**Figure 5:**
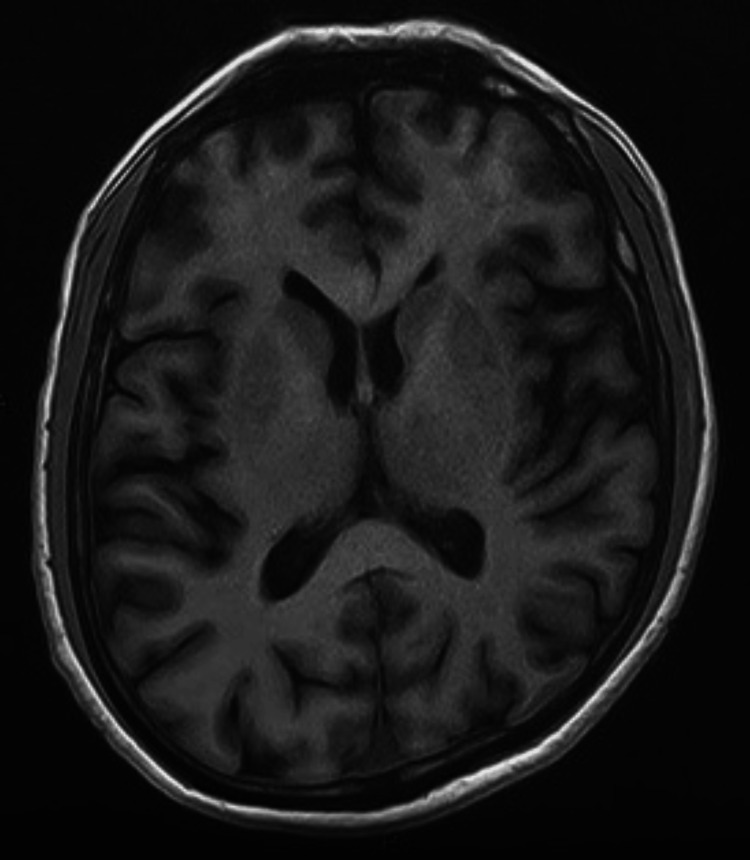
T1-weighted axial MRI plain study of the brain at the level of basal ganglia not showing any abnormality

**Figure 6:**
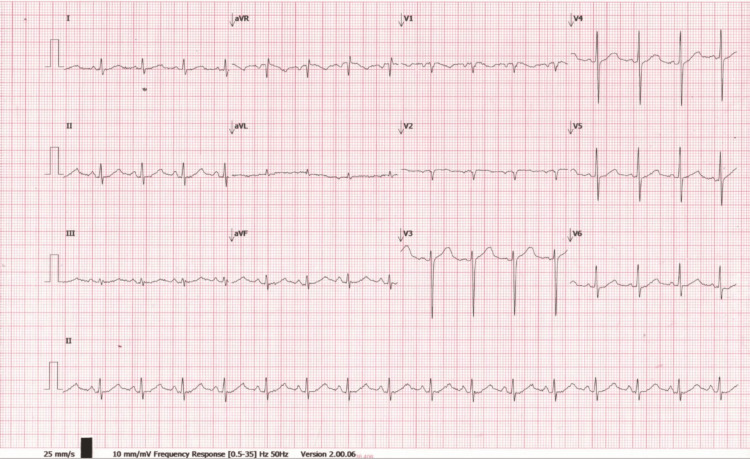
ECG all leads showing QTc 448 ms

CT of the brain was normal (Figure 4). MRI of the brain showed a possible lacunar infarct (Figure 5). Chest X-ray was normal, and abdominal ultrasound revealed mild fatty liver. ECG showed sinus rhythm with QTc 448 ms (Figure 6).

Other Tests

Troponin and awake electroencephalogram were normal.

Diagnosis and Treatment

The combination of hypokalemia, hypomagnesemia, hyponatremia, hypocalciuria, and metabolic alkalosis was suggestive of GS. The seizure was attributed to hypomagnesemia and low ionized calcium. The *SLC12A3* gene mutation test was not performed due to financial reasons. Treatment included potassium-sparing diuretics, magnesium supplements, and a diet rich in sodium, potassium, and magnesium, with regular follow-up advised. However, the patient was lost to follow-up.

## Discussion

GS typically presents with hypokalemia, hypomagnesemia, hypocalciuria, and metabolic alkalosis due to SLC12A3 gene mutations impairing NCC function in the distal convoluted tubule [2]. Both cases in this series exhibited these hallmarks but also presented with seizures, a rare manifestation, and distinct atypical features [5].

In case 1, hypocalcemia, hyperphosphatemia, low vitamin D3, secondary hyperparathyroidism, and basal ganglia calcification were unusual. Hypocalcemia likely resulted from low vitamin D3 due to limited sun exposure, compounded by hypomagnesemia impairing 1-alpha-hydroxylase activity, reducing active 1,25-dihydroxyvitamin D [6,4]. Secondary hyperparathyroidism ensued, and basal ganglia calcification may reflect chronic hypocalcemia or hypomagnesemia, though the mechanism is unclear [7]. Literature reports rare GS cases with hypocalcemia, but this constellation is novel [7].

Case 2 presented with hyponatremia, less commonly reported in GS, possibly due to chronic sodium loss and volume depletion activating the renin-angiotensin-aldosterone system (RAAS), as also evidenced by elevated renin and aldosterone in case 1 [8,3]. Seizures in both cases likely stemmed from hypomagnesemia and low ionized calcium, which prolong the QT interval and lower the seizure threshold by affecting neuronal excitability [9].

Differential diagnoses for case 1 included pseudohypoparathyroidism (ruled out by elevated PTH) and chronic kidney disease (excluded by normal glomerular filtration rate) [6]. For case 2, Bartter syndrome was considered but less likely due to hypocalciuria and the absence of childhood onset [5]. Genetic testing in case 1 was negative for homozygous *SLC12A3* mutations, suggesting possible heterozygous mutations or other genetic variants, as clinical diagnosis remains valid in such scenarios [10].

Treatment in both cases targeted electrolyte correction. Magnesium and potassium supplementation, along with vitamin D and calcium, in case 1 resolved symptoms rapidly [4]. In case 2, dietary recommendations and potassium-sparing diuretics aimed to mitigate chronic losses [3]. Long-term monitoring is essential due to risks of chondrocalcinosis and potential renal damage from chronic hypokalemia or renin-angiotensin-aldosterone activation [8,9].

## Conclusions

This case series underscores the remarkable clinical heterogeneity of GS, reminding clinicians that what is often dismissed as benign electrolyte imbalance may mask a genetically rooted and potentially debilitating condition. The presence of seizures, profound hypocalcemia, secondary hyperparathyroidism, and basal ganglia calcification - features atypical for classical GS - challenges the narrow diagnostic framework traditionally associated with this disorder. Our findings advocate for a broader diagnostic lens, especially in patients presenting with neuromuscular symptoms and refractory electrolyte disturbances. Normal renal function does not preclude a diagnosis of GS, and the absence of biallelic *SLC12A3* mutations should not delay treatment in clinically suggestive cases. These cases also highlight the importance of assessing and correcting not only potassium and magnesium but also calcium and vitamin D status, as their interplay may significantly influence neuromuscular stability and neurological outcomes. In an era where precision medicine is increasingly emphasized, these cases call for deeper genetic, biochemical, and neuroimaging exploration in unexplained seizure disorders. Ultimately, recognizing the expanded clinical spectrum of GS is not merely academic - it is critical to preventing misdiagnosis, avoiding unnecessary antiepileptic therapy, and restoring quality of life through targeted electrolyte correction.
